# The Influence of Age on the Myopic Control Efficiency of Dual-Focus Contact Lens: A Retrospective Cohort Study

**DOI:** 10.7759/cureus.97474

**Published:** 2025-11-21

**Authors:** Chia-Yi Lee, Chao Kai Chang, Ching-Yao Huang, Shun-Fa Yang, Jing-Yang Huang, Yu-Ling Chang

**Affiliations:** 1 Institute of Medicine, Chung Shan Medical University, Taichung, TWN; 2 Eye Clinic, Nobel Eye Institute, Taipei, TWN; 3 Department of Optometry, Da-Yeh University, Changhua, TWN; 4 Department of Medical Research, Chung Shan Medical University Hospital, Taichung, TWN; 5 Department of Pediatric Medicine, Cathay General Hospital, Taipei, TWN

**Keywords:** age, axial length, dual-focus contact lens, myopia, spherical equivalent refraction

## Abstract

Purpose: The purpose of this study was to survey the effectiveness of dual-focus contact lenses (DFCLs) in controlling myopia in children of different ages.

Method: A retrospective cohort study was conducted, and individuals receiving DFCL treatment were included. Then, the study populations were classified according to age, comprising a total of 62 and 94 eyes in the young (younger than 12 years old) and old groups (older than 12 years old), respectively. The primary outcomes of our study were changes in spherical equivalent refraction (SER) and axial length (AXL). The independent t-test and generalized linear model were applied for the statistical analyses.

Results: The baseline SERs were -2.55±0.71 diopter (D) and -2.69±0.77D in the young and old groups. At the one-year follow-up, the SERs were -2.87±0.83D and -2.83±0.85D in the young and old groups, and SER progression was significantly higher in the young group than the old group (p = 0.002). The initial AXLs were 24.69±0.44 mm and 24.75±0.50 mm in the young and old groups, respectively, and 24.83±0.52 mm and 24.85±0.55 mm at the final visit. The AXL elongation was significantly higher in the young group compared to the old group (p < 0.001). The high near-work activity was associated with higher SER and AXL changes in both groups (both p < 0.05), and the high outdoor activity was associated with lower SER and AXL changes in both groups (both p < 0.05).

Conclusions: Old age correlates with better myopic control regarding SER and AXL changes for DFCL users.

## Introduction

Myopia is a growing health problem in many regions, and its prevalence may reach more than 75% in Asian countries [1]. The development of myopia is associated with both a steep corneal curvature and long axial length (AXL), and the latter is the main etiology of acquired myopia [2,3]. High myopia, largely defined by a spherical equivalent refraction (SER) of more than -6.00 diopters (D), correlates with a higher possibility of insults to the retinal pigment epithelium, neurosensory retina, and optic nerve [4]. Several complications of high myopia, such as retinal detachment and myopic choroidal neovascularization, can result in severe visual impairment [3].

Because of the impact of myopia on the eye, several interventions have been developed to prevent deterioration [5,6]. Topical atropine instillation may be the most widely utilized myopia intervention in the world [7]. In a previous publication, the administration of atropine significantly controlled the progression of both AXL and SER [8,9]. In addition to topical atropine, orthokeratology contact lenses have been applied to control the progression of myopia with good efficiency and safety [1,10]. In addition to the above two interventions, the defocus-incorporated multiple-segment spectacle lens was introduced during the 2010s, and its effect in controlling myopia was also acceptable [11].

The dual-focus contact lens (DFCL) is a daily disposable contact lens that has been in use for the control of myopia for more than five years [12]. In earlier studies, the myopic control effect of DFCL regarding SER and AXL was shown to be excellent [13,14]. However, there are few studies investigating the myopic control effect of DFCL in populations of different ages, despite age being a risk factor for the progression of myopia [15,16]. In addition, lifestyle factors could alter the effectiveness of myopic control [5], and whether there exists an interaction between DFCL usage and lifestyle factors requires further study.

Accordingly, the purpose of our study is to evaluate the myopic control effect of DFCL through changes in SER and AXL in patients of different ages. The correlations between lifestyle factors and the myopic control effect of DFCL were also analyzed.

## Materials and methods

Ethical declaration

All the interventions mentioned in our study obeyed the 1964 Declaration of Helsinki and its consecutive amendments. Moreover, our study was approved by the Institutional Review Board of the National Changhua University of Education (project code: NCUEREC-112-071; date of approval: 27 September 2023). The requirement for written informed consent was waived by the National Changhua University of Education as a result of the retrospective design of our study.

Patient selection

Our retrospective cohort study was carried out at the Nobel Eye Institute, which is a group of over 20 clinics in Taipei, Taiwan. Individuals who met the following criteria were included in the study population: (1) aged from 9 to 18 years old, (2) received DFCL management at any branch of the Nobel Eye Institute, and (3) attended follow-up appointments at any branch of the Nobel Eye Institute at least six times over one year. The purpose we set the six follow-up criteria is to ensure the children visit our clinics regularly. One type of DFCL (MiSight, CooperVision, Victor, NY) was given to all the individuals in our study. The individuals were asked to continuously wear the DFCL for 10-12 hours per day, and there was no “holiday” for the DFCL. After the removal of the DFCL, individuals wore single-vision spectacles to gain fair vision during the remaining time. The following exclusion criteria were applied in our study to standardize the ocular condition of the study population: (1) a corrected distance visual acuity (CDVA) below 20/25; (2) a high myopia status, i.e., an SER of more than -6.00D at initial presentation; (3) prominent ocular diseases, including but not limited to corneal opacity, corneal neovascularization, infantile glaucoma, congenital cataract, optic nerve atrophy, retinopathy of prematurity, retinal detachment, juvenile idiopathic arthritis uveitis, eyeball rupture, and microophthalmos; and (4) application of additional myopic control therapy other than DFCL management (i.e., topical atropine treatment) during the follow-up period. In the next step, the individuals were divided into a younger group (younger than 12 years old) and an older group (older than 12 years old). We used 12 years old as the cut-point because children in Taiwan reach puberty at around this age [17]. Of note is that only the right eye of each individual was included in our study. Finally, there were a total of 62 and 94 eyes in the young and old groups, respectively. We did not match the two groups into the same number because we want to include as many eyes as possible.

Myopia examination

All the individuals at our institute received the same DFCL examination protocol, and all the medical information for each participant in our study was obtained from their medical records. The pre-treatment data included the CDVA, sphere power and cylinder power, SER, flat and steep keratometry, and AXL. The CDVA was obtained via the Snellen chart, and the sphere power and cylinder power were obtained after cycloplegia using an autorefractor (KR-8900, Topcon, Itabashi-ku, Tokyo, Japan). To perform the cycloplegia procedure, topical tropicamide (Better eye drop; Aseptic Innovative Medicine Co., Ltd., Taoyuan, Taiwan) was dropped into the eye at least three times, and optometrists evaluated the pupil diameter. If the pupil diameter after cycloplegia was more than 8 mm, three cycloplegia refraction measurements were performed using an autorefractor, and the average value was recorded. The cycloplegia SER in our study was defined as the sphere power plus half of the cylinder power. Both flat and steep keratometry were measured via a topographic instrument (TMS-5; Tomey Corporation, Nishi-Ku, Japan), and the AXL was measured with a biometry instrument (IOL Master 500; Carl Zeiss, Göschwitzer Str., Jena, Germany). After the usage of DFCL, the post-treatment examination included measurement of the CDVA, cycloplegia refraction, and AXL. The primary outcomes in our study were the changes in SER and AXL; for this, the values of SER and AXL before and one year after the DFCL management were obtained and used for the statistical analysis.

Statistical analysis

Statistical Product and Service Solutions (SPSS, version 20; IBM SPSS Statistics for Windows, Armonk, NY) was employed for statistical analyses in our study. The Shapiro-Wilk test was employed to check the normality of the two study groups, and both groups showed normal distributions (p > 0.05), with a statistical power of 0.93, alpha value of 0.05, and medium effect size obtained via G∗power (version 3.1.9.2; The G*Power Team, Germany). There was no missing data or outliers in our study. Descriptive analysis was employed for the presentation of baseline data of the two groups, and the chi-square test and the independent T-test were employed to compare the differences in baseline data between the young and old groups. The choice of the chi-square test or the independent T-test in the above analysis was dependent on the features of the baseline data. The independent T-test was employed to compare the amount of SER progression and AXL elongation in different time periods between the young and old groups. In the next step, the generalized linear model was employed to analyze the correlation between specific parameters and SER progression and AXL elongation in the two groups separately. These specific parameters included male sex, high initial AXL (more than 25 mm), high near-work activity (spending more than three hours per day on near-work activities such as reading or using a cellphone after school), and high outdoor activity (spending more than one hour on outdoor activity in one day). The odds ratio (OR) and corresponding 95% confidence interval (CI) of each parameter for the changes in SER or AXL were calculated via the generalized linear model. Statistical significance was defined as p < 0.05, and a p value lower than 0.001 was presented as p < 0.001.

## Results

The baseline data of the two groups are presented in Table 1. The mean ages in the young and old groups were 10.62±0.67 and 14.21±0.86, respectively. The difference in age between the two groups was significant as a result of our classification strategy (p < 0.001). However, there were no significant differences in sex distribution between the two groups (p = 0.836). In terms of the ophthalmic parameters, there were no significant differences in the CDVA, sphere power, cylinder power, or SER between the young and old groups (all p > 0.05). In addition, both flat and steep keratometry and AXL demonstrated similar values between the young and old groups (all p > 0.05) (Table 1).

**Table 1:** Baseline characteristics of the two groups. AXL: axial elongation; CDVA: corrected distance visual acuity; D: diopter; N: number; SER: spherical equivalent refraction * denotes a significant difference between the two groups.

Characters	Young group (N= 62)	Old group (N= 94)	P
Age (year)	10.62±0.67	14.21±0.86	<0.001*
Sex (male: female)	26:36	41:53	0.836
CDVA (LogMAR)	0.00±0.01	0.00±0.00	0.999
Sphere power (D)	-2.28±0.87	-2.39±0.95	0.466
Cylinder power (D)	-0.54±0.23	-0.60±0.26	0.142
SER (D)	-2.55±0.71	-2.69±0.77	0.254
Flat keratometry (D)	42.33±1.58	42.10±1.73	0.402
Steep keratometry (D)	43.09±1.69	42.98±1.93	0.715
AXL (mm)	24.69±0.44	24.75±0.50	0.443

The baseline SERs were -2.55±0.71D and -2.69±0.77D in the young and old groups, respectively. At the one-year follow-up, the SER was -2.87±0.83D and -2.83±0.85D in the young and old groups, respectively. The SER progression was significantly higher in the young group than in the old group (p = 0.002) (Table 2). The initial AXLs were 24.69±0.44 mm and 24.75±0.50 mm in the young and old groups, respectively, and 24.83±0.52 mm and 24.85±0.55 mm at the final visit. The AXL elongation was significantly higher in the young group compared to the old group (p < 0.001) (Table 2).

**Table 2:** Progression of spherical equivalent refraction and axial length after the follow-up interval. AXL: axial elongation; D: diopter; N: number; SER: spherical equivalent refraction * denotes a significant difference between the two groups.

Index	Young group	Old group	P
SER (D)	-	-	-
Pre-treatment	-2.55±0.71	-2.69±0.77	0.254
Post-treatment	-2.87±0.83	-2.83±0.85	0.772
Increment	-0.32±0.17	-0.24±0.14	0.002*
AXL (mm)	-	-	-
Pre-treatment	24.69±0.44	24.75±0.50	0.443
Post-treatment	24.83±0.52	24.85±0.55	0.821
Increment	0.14±0.06	0.10±0.04	<0.001*

Concerning the correlation between specific parameters and the myopic progression in different groups, the high initial AXL and high near-work activity were associated with greater changes in SER and AXL in the young group, while high outdoor activity was associated with lower changes in SER and AXL in the young group (all p < 0.05) (Table 3). However, the high initial AXL correlated with greater changes in AXL, while high near-work activity correlated with greater changes in SER and AXL in the old group (all p < 0.05). In addition, high outdoor activity correlated with lower changes in SER and AXL in the old group (all p < 0.05) (Table 4).

**Table 3:** Risk factors for myopic progression in the young group. AXL: axial elongation; CI: confidence interval; OR: odds ratio; SER: spherical equivalent refraction * denotes a significant correlation between the parameter and myopic progression.

Parameter	OR	95% CI	P
SER	-	-	-
Male sex	0.993	0.982-1.004	0.448
High initial AXL	1.025	1.002-1.047	0.030*
High near-work activity	1.028	1.006-1.050	0.001*
High outdoor activity	0.972	0.965-0.980	<0.001*
AXL	-	-	-
Male sex	0.995	0.984-1.006	0.567
High initial AXL	1.017	1.004-1.031	0.026*
High near-work activity	1.023	1.004-1.043	0.005*
High outdoor activity	0.979	0.969-0.990	<0.001*

**Table 4:** Risk factors for myopic progression in the older group. AXL: axial elongation; CI: confidence interval; OR: odds ratio; SER: spherical equivalent refraction * denotes a significant correlation between the parameter and myopic progression.

Parameter	OR	95% CI	P
SER	-	-	-
Male sex	0.991	0.982-1.001	0.073
High initial AXL	1.010	0.998-1.022	0.105
High near-work activity	1.016	1.003-1.029	0.012*
High outdoor activity	0.984	0.972-0.996	0.006*
AXL	-	-	-
Male sex	0.994	0.980-1.009	0.672
High initial AXL	1.012	1.002-1.022	0.037*
High near-work activity	1.008	1.001-1.015	0.045*
High outdoor activity	0.990	0.982-0.998	0.040*

## Discussion

In our study, young age was associated with greater progressions in SER and AXL than in older individuals when receiving DFCL treatment. Moreover, near-work activity and outdoor activity can significantly influence the degree of myopic progression in populations of different ages with DFCL management. However, the initial AXL is mainly correlated with myopic progression in young children receiving DFCL treatment.

Both general myopia and high myopia can result in several ocular disorders according to a previous study [2]. Myopic maculopathy presents a disruption of Bruch’s membrane, as well as the outer retinal layer [3]. In severe forms of myopic maculopathy, choroidal neovascularization can develop, in which the intravitreal injection of anti-vascular endothelial growth factor may be warranted to protect vision [2,3]. Despite prompt treatment, the outcomes of myopic maculopathy are poor, and prominent visual impairment may still occur [18]. Retinal detachment is another negative complication of both myopia and high myopia [3]. Rhegmatogenous retinal detachment emerges more frequently in individuals with higher myopia, with one study finding a 200-fold higher chance of rhegmatogenous retinal detachment in such individuals compared to the general population [2]. Surgical interventions, including pneumatic retinopexy, scleral buckling, and trans pars plana vitrectomy, may be advocated to manage rhegmatogenous retinal detachment, but the postoperative visual performance may not always be positive [19]. In addition to retinal diseases, glaucoma is another common eye disease in individuals with high myopia that may contribute to an irreversible deterioration in visual ability [3,20]. As a consequence, the importance of preventing the deterioration of myopia and the occurrence of high myopia cannot be overemphasized. At present, several tools for controlling myopia have been introduced with acceptable ability, including atropine eyedrops, orthokeratology contact lenses, DIMS spectacle lenses, and DFCLs [21]. Despite the efficacy of these methods in the general population, their success in specific populations has not been evaluated thoroughly. We suspect that tools for controlling myopia, such as DFCLs, may demonstrate different efficacies in individuals from different demographics. This hypothesis is supported by the analyses of our study.

The application of DFCLs illustrates better control of SER and AXL in older individuals compared to younger participants. In an earlier publication, DFCLs demonstrated excellent myopic control efficiency in individuals of different ages [12]. In addition, the combined application of DFCLs and atropine eyedrops yielded a good myopic control effect in a population of 7-13 years old [22]. Nevertheless, there is limited research investigating the degree of myopic control of DFCLs in populations of different ages. Our findings represent preliminary evidence of greater myopic control efficiency of DFCLs in individuals aged 12 years or older than in individuals aged younger than 12. In addition, the baseline data of the two groups in our study, including CDVA, SER, and AXL, were similar; thus, the influence of these crucial parameters may be minor. Furthermore, no individuals receiving any other myopia control tool than DFCL management were enrolled in our study, so the potential influence of tools was excluded. Consequently, being aged over 12 years may be independently correlated with a better success rate of DFCLs in controlling myopia. In terms of research investigating the relationship between other myopia control tools and age, a previous publication presented a better myopia control effect of orthokeratology contact lenses in older individuals [23]. Combining the results of that study and ours, it is possible that contact lens-based myopia control interventions are more effective at preventing the progression of myopia in the senior population. Future work should aim to find the exact etiology for this phenomenon. Although the young group in our study demonstrated a poorer myopia control efficiency than the older group, the SER progression of the young group was only around -0.30D after one year of usage. As a consequence, the myopia control efficiency of the DFCL intervention could still be adequate for the young population. Still, the additional myopic control tool, such as atropine eyedrop, may be considered for children younger than 12 years with DFCL application, and further research is warranted to survey the effect of combined DFCL and other myopic control tools on the population that is younger than 12 years.

Regarding the sensitivity analysis, near-work activity is associated with a greater progression of SER and AXL in both groups, while outdoor activity correlates with better control of SER and AXL. In earlier research, near-work activity was a significant risk factor for myopic progression [5]. The increase in near-work activities has been associated with a significantly higher likelihood of developing and deteriorating myopia [24]. On the other hand, the performance of outdoor activity is a known protective factor against the development and progression of myopia [6]. Children who perform an outdoor activity for more than six hours per week experience a significantly lower risk of developing myopia [25]. Furthermore, individuals who perform an outdoor activity during recess demonstrated a lower incidence of myopia in an Asian community [26]. By providing adequate outdoor activity, the rates of myopic development and progression were decreased compared to other populations of the same ethnicity from the same residential area [27]. Our findings further correspond to the preceding conclusions and highlight the importance of lifestyle modulation for the control of myopia, even with the use of good myopic control management tools, such as DFCLs. The high initial AXL is correlated with greater progression of myopia in our study, while SER progression is lower in the older group. A possible explanation for this phenomenon may be a lower tendency for myopic progression in the older population [15], and the mild difference in changes in amplitude between SER and AXL may have influenced the analysis results. The correlation between a high initial AXL and high AXL progression in the older group indicates that the influence of high initial AXL on myopic progression in this age group could be as prominent as in the young group.

Concerning the effect of myopia control in our study, the mean SER progressions after one year of DFCL treatment were -0.32D and -0.24D in the young and old groups, respectively. In previous studies using DFCLs to control myopia, the mean annual SER progression ranged from -0.17D to -0.42D, which is comparable to the SER results obtained in our study [12-14]. In terms of the amplitude of AXL elongation, the mean values after one year of DFCL treatment were 0.14 mm and 0.10 mm in our study. In previous studies controlling AXL with DFCL treatment, the mean annual AXL elongation was around 0.10-0.16 mm [12-14]. Consequently, the AXL control efficiency in our study is similar to the earlier publications. Furthermore, the SER and AXL progressions in our study are comparable to those of previous studies that applied other types of myopic control interventions [28-30]. As a consequence, the efficacy and quality of the DFCL application for myopic control in our institutes could be acceptable.

There were some limitations in our study. First, the postoperative keratometry was not measured because we do not routinely measure it in clinical practice and the design of our study is retrospective. In addition, the total number of eyes in our study is relatively low; only 156 eyes were enrolled, which may contribute to statistical bias, although the normality test and statistical power exam demonstrated acceptable values. In addition, the follow-up period of our study was only one year, which might not be adequate; in comparison, one previous study tracked individuals receiving DFCL management for six years [13]. Additionally, we applied tropicamide, rather than homatropine, for the cycloplegia procedure since we did not own homatropine in our institution. Thus, the accuracy of the cycloplegia procedure may be influenced due to the weak cycloplegia effect of tropicamide, although the pupil diameter was checked before the cycloplegia refraction measurements. Finally, all the children selected in our study were Han Taiwanese; thus, the external validity of our study may be low.

## Conclusions

In conclusion, an age of more than 12 years correlates with a better myopic control effect of the DFCL intervention. Furthermore, lifestyle factors can have a significant effect. Consequently, additional tools such as atropine for the control of myopia and lifestyle modifications may be recommended for younger DFCL users. Further large-scale prospective studies exploring the optimal adjuvant therapy for myopic control in young DFCL users are mandatory.
